# Influence of calcium chloride impregnation on the thermal and high-temperature carbonization properties of bamboo fiber

**DOI:** 10.1371/journal.pone.0212886

**Published:** 2019-02-28

**Authors:** Dali Cheng, Tao Li, Gregory Smith, Jing Yang, Cheng Hang, Zhenyue Miao, Zicheng Wu

**Affiliations:** 1 College of Materials Science and Engineering, Nanjing Forestry University, Nanjing, China; 2 Department of Wood Science, University of British Columbia, Vancouver, Canada; 3 State Key Laboratory of Coordination Chemistry, Nanjing University, Nanjing, China; 4 Anhui Product Quality Supervision and Inspection Institute / National Testing Center for Funtional Fiber & Textile, Hefei, China; Helsingin Yliopisto, FINLAND

## Abstract

In this study, bamboo fiber was pretreated with calcium chloride (CaCl_2_) solution by using an ultrasonic method, and then heat-treated at 250°C and carbonized at 1000°C. The effect of impregnation with CaCl_2_ on the thermal and chemical properties and morphology of bamboo fiber was determined using thermogravimetric and differential thermogravimetric analyses, in situ Fourier transform infrared spectroscopy, and scanning electron microscopy. The pore structure of the carbonized bamboo fiber was investigated. The results revealed that bamboo fiber pretreated with 5% CaCl_2_ (BFCa_5_) showed a downward shift in the temperature of the maximum rate of weight loss253°C and increase in char residue to 31.89%. BFCa_5_ was expected to undergo dehydration under the combined effect of oxygen-rich atmosphere and CaCl_2_ catalysis from 210°C, and cellulose decomposition would be remarkable at 250°C. Pretreatment with 5% CaCl_2_ promoted the formation of porous structure of the carbonized fiber, which exhibited a typical Type-IV isotherm, with the Brunauer–Emmett–Teller specific surface area of 331.32 m^2^/g and Barrett–Joyner–Halenda adsorption average pore diameter of 13.6440 nm. Thus, CaCl_2_ was found to be an effective catalyst for the pyrolysis of bamboo fiber, facilitating the formation of porous carbonized fiber.

## Introduction

In recent years, biomass-based products from naturally renewable resources have been attracting considerable attention. Cellulose-based carbon fibers and corresponding reinforced composite materials are good examples of materials developed using renewable resources such as bamboo [1, 2]. Bamboo has several advantages over other plant fibers such as low density; low cost; high mechanical strength, stiffness, and growth rate; and ability to fix atmospheric carbon dioxide [3, 4]. In China, bamboo, especially Moso Bamboo, is one of the most important and abundant resources of fibers having relatively high mechanical strength. Bamboo-derived rayon fiber is a new promising environmental fabric material that has gained acceptance for manufacturing and processing textiles. It was named “Chinese Fiber” because of its good strength, wear resistance, and flexibility. These fibers are non-toxic, non-carcinogenic, biocompatible, and non-toxic to the biological environment.

More than 80% of cellulose- or rayon-based carbon fibers are known to shrink and lose weight after carbonization [5]. Further, the carbon yield of these fibers is relatively low. Byrne et al. [6] found that anhydrosaccharides were important intermediates during the pyrolytic degradation of cellulose. Huang and Li [7] extensively investigated the thermal degradation of cellulose and found that the macromolecular structure of cellulose plays an important role in changing its thermal stability. Kawamoto et al. [8] determined the cellulose pyrolysis mechanism and concluded that levoglucosan is the primary direct product of cellulose decomposition, and it is consequently degraded to low-molecular-weight products or polymerized into polysaccharides that become carbonized, leading to the formation of char. There is competition between the formation of low-molecular-weight products and solid char. However, the yields of volatile tarry products containing levoglucosan would adversely affect the quality of the carbonized fibers formed. Therefore, preparing high-quality rayon-based carbon fibers is necessary by effectively controlling the reaction pathways in the initial stage of fiber pyrolysis. Pretreating biomass or cellulose with phosphoric acid or alkaline and inorganic salts can accelerate the pyrolysis pathway reactions, in which more cellulose would decompose to low-molecular-weight fragments instead of levoglucosan. [9,10]. Kleen and Gellerstedt [11] concluded that calcium ions increased the yield of anhydrosugars during pulp pyrolysis. In a recent study, Shimada et al. [12] reported that both alkali and alkaline earth metals significantly influenced the formation of low-molecular-weight compounds. Moreover, as compared to alkalis, alkaline earth metals extremely reduced the bulk cellulose decomposition temperature. Varhegyi et al. [13] found that the onset temperature of decomposition of cellulose pyrolysis and maximum weight loss were decreased when cellulose was impregnated with inorganic compounds such as NaCl, MgCl_2_, and ZnCl_2_. Numerous studies have investigated the influence of transition metal salts on the pyrolysis of wood and cellulose [14].

The effectiveness and influence of CaCl_2_ impregnation on the thermal and high-temperature carbonization properties of bamboo fibers have not yet been investigated in detail. More attention needs to be paid to the chemical change of bamboo fiber after impregnation in thermal environment and its microstructure analysis after carbonization. In this study, the thermal characterization of bamboo fibers treated with CaCl_2_ was performed using thermo gravimetric analysis (TGA). Further, in situ Fourier transform infrared spectroscopy (FTIR) measurement was used to analyze directly the changes in cellulose structure during heat treatment. Scanning electron microscopy (SEM) was used to investigate the chemical and morphology changes in bamboo fibers pretreated with CaCl_2_ after heat treatment with increasing temperature, and high-temperature carbonization and the pore structure of carbonized bamboo fibers was also investigated.

## Materials and methods

### Materials

Bamboo-derived rayon fibers (hereafter bamboo fiber), a kind of regenerated cellulose fiber having cellulose II structure, were collected from Zhejiang, China. The polymerization degree of the bamboo fibers is 350–380, and they contain less than 0.2% ash. The weight percentages of C, H, and N in the fibers were measured by an elemental analyzer (FLASH 2000 Series Elemental Analyzer, Thermo Fisher Scientific Inc.USA) to accomplish the elemental analysis. The weight percentage of O was obtained by difference, that means, O (wt%) = 100-C-H-N. Elemental analysis data of bamboo fiber after washing is shown in Table 1. Impurities in the fiber samples could affect experimental results; hence, the bamboo fibers were washed with warm distilled water (45°C), and the conductivity of the washed water was measured. Washing was continued until the conductivity of the washed water was the same as that of distilled water. Then the washed fiber was dried in a vacuum drying oven at 80°C for 24h.

**Table 1:** Elemental composition of bamboo fiber.

	C (wt.%)	H (wt.%)	O (wt.%)	S (wt.%)	N (wt.%)
Bamboo fiber	44	6.53	49.309	0.072	0.089

### Ethics statement

The bamboo fibers collected for this study are not threatened species. Moso bamboo we selected is situated between 30°23’ N to 30°53’ N and 119°14’E to 119°53’ E. There was no specific permissions were required for the location. The sites in this study is common for bamboo shoot and culm production. The field studies did not involve endangered or protected species. The conducted research is in compliance with laws and ethical standards of the countries in which research was conducted.

### Pretreatment of bamboo fibers and carbonized fibers

The CaCl_2_ reagent used was of analytical grade (>99.0%) and was obtained from Nanjing Chemical Reagents Corp., P. R. China. First, different concentrations (1%, 5%, and 10% w/w) of CaCl_2_ solutions were prepared, and then the above bamboo fibers were impregnated in these solutions at a bath ratio of 1:30 (w/w); subsequently, they were placed in an ultrasonic generator (power, 500 w) for 1 h at 25°C, followed by overnight vacuum drying. Next, the fiber with the best thermal property was heat-treated at 250°C for 30 min with the heating rate of 10°C/min, the fiber was heat-treated at 600°C for 10min with the heating rate of 10°C/min. They were then carbonized at 1000°C for 3 min with the heating rate of 20°C/min. Carbonization process of bamboo fibers cured was performed by ultra-high pure argon (99.999%) using a tube-type carbonization furnace.

### Analysis method

#### TG analysis

The thermogravimetric analyzer TGA Q500 (TA instrument, USA) was used. The weight loss of bamboo fibers during pyrolysis was measured using TGA under the working atmosphere of ultra-high pure argon (99.999%). Approximately 5 mg sample was heated at room temperature up to final temperature 800°C at the heating rate of 20°C/min during pyrolysis and a steady nitrogen flow rate of 100 ml/min.

#### The in situ FTIR analysis

For spectroscopic measurements, a Nicolet FTIR Spectrometer 360 (Thermo Fisher Scientific, USA) equipped with a Specac model temperature controlling system with a high-temperature cell was used. The heating rate was 20°C/min, and the FTIR spectrum was recorded at 100°C, 150°C, 210°C, 230°C, 250°C, and 260°C. For the preparation of FTIR specimens, the fiber samples were first milled into powder; next, approximately 5 mg of powder sample was ground and uniformly mixed with 200 mg KBr powder. Before mixing, fiber sample, as well as KBr, was dried under an infrared lamp for 30 min.

#### Morphology and structure analysis

The morphology and structure of the bamboo fibers were determined using scanning electron microscopy (SEM; FEI Quanta 200). The N_2_ adsorption/desorption was performed at -196°C by using Micromeritics ASAP 2020 to determine the pore structure and specific surface area of the carbonized fiber samples. The completely activated samples were obtained by heating them at 80°C under dynamic high vacuum for 10 h. The specific surface area was calculated using the multiple-point Brunauer–Emmett–Teller (BET) model. Porosity distributions were obtained from the N_2_ adsorption/desorption isotherms, which were used to identify a dominant pore size, by using the Barrett–Joyner–Halenda (BJH) method [15].

## Results and discussion

### The thermal characterization of bamboo fiber

**Fig 1:**
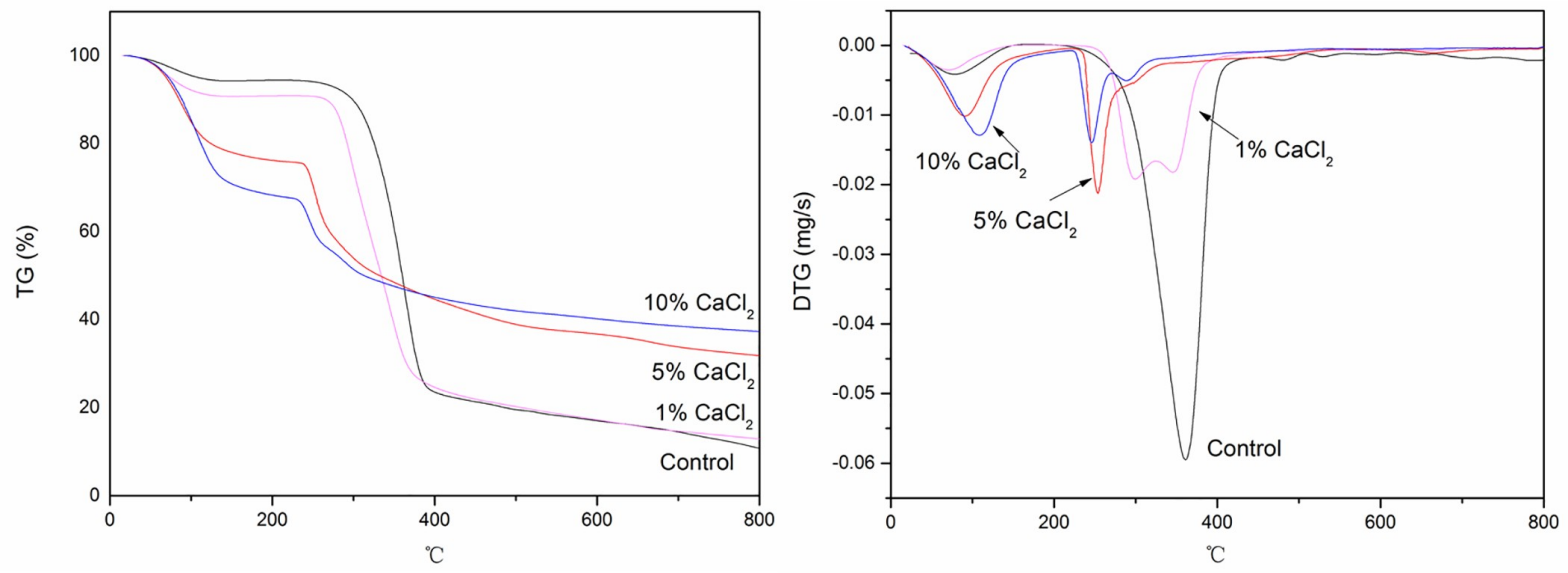
The thermogravimetric (TG) and differential thermogravimetric (DTG) curves of the control and bamboo fibers treated with CaCl2.

The dynamic gravimetric (TG and DTG) curves of untreated bamboo fiber (designated as control) and bamboo fiber impregnated with different concentrations of CaCl_2_ (denoted BFCa) are shown in Fig 1.

Comparing the mass loss of the control with the treated samples, one can see that the presence of CaCl_2_ inhibits sample degradation and improves with CaCl_2_ concentration. The TG and DTG curves for the control were the typical dynamic gravimetric curves for cellulosic materials. The rate of weight loss of the control sample was higher over a relatively narrow range of temperatures from 230°C to 470°C, corresponding to a weight loss of around 74%. The char residue was about 10.80% when the temperature was increased up to 800°C. Further, the DTG curve of the control indicated that the maximum rate of weight loss occured at 364°C.

For BFCa, the temperature of the maximum rate of weight loss (T_M_)was shifted to a lower side and char residue formation increased with increasing concentration of CaCl_2_. The T_M_ was lowered to 253°C with 5% CaCl_2_ pretreatment. The DTG curve of bamboo fiber treated with 1% CaCl_2_ showed two maximums: one at 298°C and the other at 345°C, indicating that the decomposition reaction comprised two steps. The DTG curve of bamboo fibers treated with 10% CaCl_2_ also showed two maximums: one at 246°C and the other at 290°C. Furthermore, the char residue increased to 13.04%, 31.89%, and 36.92% at 800°C after pretreatment with 1%, 5%, and 10% CaCl_2_, respectively; the maximum decomposition rate of BFCa was lower than that of the control. Thus, CaCl_2_ was thought to be an effective catalyst for the decomposition of bamboo fiber. Bamboo fiber pretreated with CaCl_2_ showed lower T_M_ of less than 300°C and decreased maximum weight loss, whereas increased char residue formation. These findings can be mainly attributed to the increased release of H_2_O and CO_2_ at the expense of flammable volatiles released during pyrolysis; thus, the flammability of carbon fibers decreased. Furthermore, the increased amount of char residue could act as a thermal barrier that reduced heat transfer to some extent and prevented the outward flow of combustible gases, thereby reducing cellulose decomposition [16]. Varhegyi et al. [13] found that the temperature for the onset of decomposition and maximum rate of weight loss were decreased when inorganic compounds were present during pyrolysis, in agreement with our results. Therefore, CaCl_2_ could not only catalyze the depolymerization reaction leading to the production of macromolecules but also accelerate the degradation of these macromolecules and generating numerous smaller molecules. In addition, ultrasonic pretreatment could increase the solubility and penetration of chemical agents, and it was found to be an effective and favorable strategy to improve the activation of fibers [17]. Cellulose and regenerated cellulose treated with various inorganic compounds could catalyze the dehydration reaction, thereby effectively accelerating the pyrolysis reaction [18–20]. Therefore, although bamboo fibers treated with 10% CaCl_2_ showed the highest char residue and lowest T_M_, these were not considerably different from those for bamboo fiber treated with 5% CaCl_2_, which showed values higher than the control. Thus, 5% CaCl_2_ was chosen as the optional treatment.

### In situ FTIR analysis

**Fig 2:**
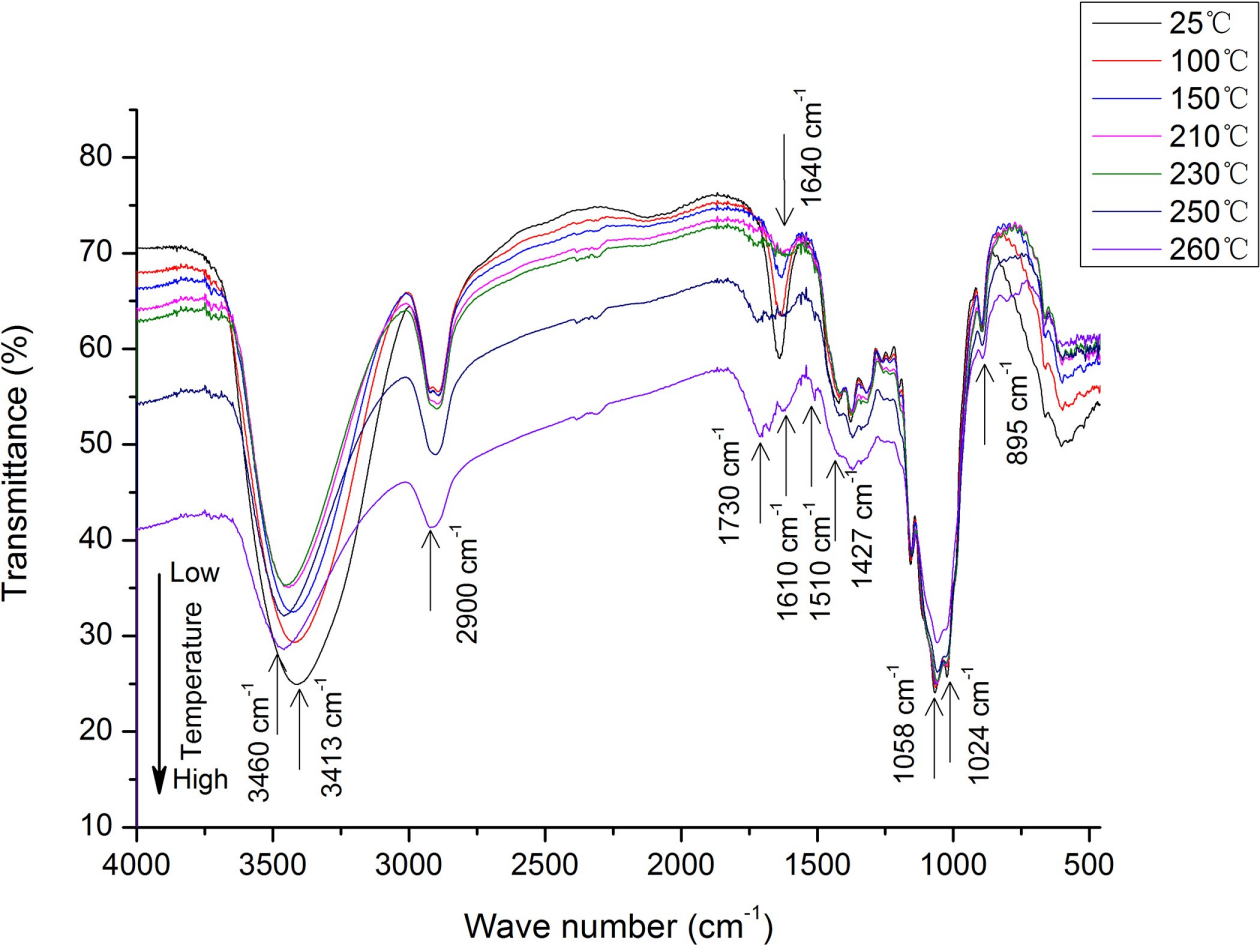
The Fourier transform infrared spectroscopy (FTIR) spectrum of bamboo fiber treated with 5% CaCl_2_ (BFCa_5_) at increasing temperature.

The FTIR spectrum of bamboo fiber treated with 5% CaCl_2_ (defined as BFCa_5_) at different temperatures is shown in Fig 2.

The intramolecular hydrogen bonds of O(2)H…O(6) and O(3)H…O(5) and the intermolecular hydrogen bond of O(6)H…O(3) in cellulose are generally present at 3455–3410, 3375–3340, and 3310–3230 cm^-1^, respectively [21,22]. When the temperature was increased to 260°C, the maximum absorbance of the OH stretching vibration was shifted to a higher wavelength at 3460 cm^-1^, showing intramolecular hydrogen bonding of O(2)H…O(6) to be decreased by dehydration and even more by subsequent thermaltreatment. The band at around 2900 cm^-1^, the main intensity of which is attributed to the CH stretching vibrations, decreased gradually with increasing temperature.

The sharp peak appearing at 1640 cm^-1^ in BFCa_5_ samples can be attributed to the bending mode of the absorbed water in cellulose. The peak intensity began to decrease when the temperature was raised to 100°C; cellulose is expected to undergo dehydration under the combined effect of oxygen-rich atmosphere and CaCl_2_ catalysis when the temperature is continued to increase to 210°C.

The existence of an adsorption peak at 1730 cm^-1^, which is represented by C = O vibrations corresponding to carbonyl, ester, or carboxyl bonds, is caused by the dehydration of water from the equatorial hydroxyl groups in monomers [23]. The intensity of the adsorption peak would increase with increasing temperature; cellulose could also undergo dehydration accompanied by the formation of various unsaturated C = C bonds or enolic groups [24, 25].

The characteristic adsorption peaks at 1024, 1162, and 1427 cm^-1^ represented C-O vibrations of secondary alcohol [26], C-O-C stretching at the β-(1–4)-glycosidic linkage [27, 28], and CH_2_ bending [28], respectively. Notably, the absorbance of these bands was reduced with increasing temperature, and the band at 1427 cm^-1^ disappeared when the temperature reached more than 250°C. Moreover, the intensity of the bands at 1024 and 1162 cm^-1^ decreased remarkably at 250°C and disappeared when the temperature reached 260°C. This indicates that cellulose decomposition would be remarkable at 250°C, which is consistent with the findings of TG/DTG analysis. The subsequent reactions include polymerization or condensation, which might be induced by intermolecular dehydration. The aromatization of polymers also occurs simultaneously, and the peaks at 1610 cm^-1^ and 1510 cm^-1^ vibrations were detected with increasing heat treatment when the temperature reached 250°C, which represents the skeleton stretching vibrations of the benzene ring. Aromatic clusters might be produced by the condensation of the aromatized molecules generated during the decomposition/dehydration of oligosaccharides or monosaccharides [29].

### Morphology and structure analysis

Physical and chemical activation processes are well-known strategies to produce highly porous carbon materials from coal-derived precursors or organic compounds [30–32].

**Fig 3:**
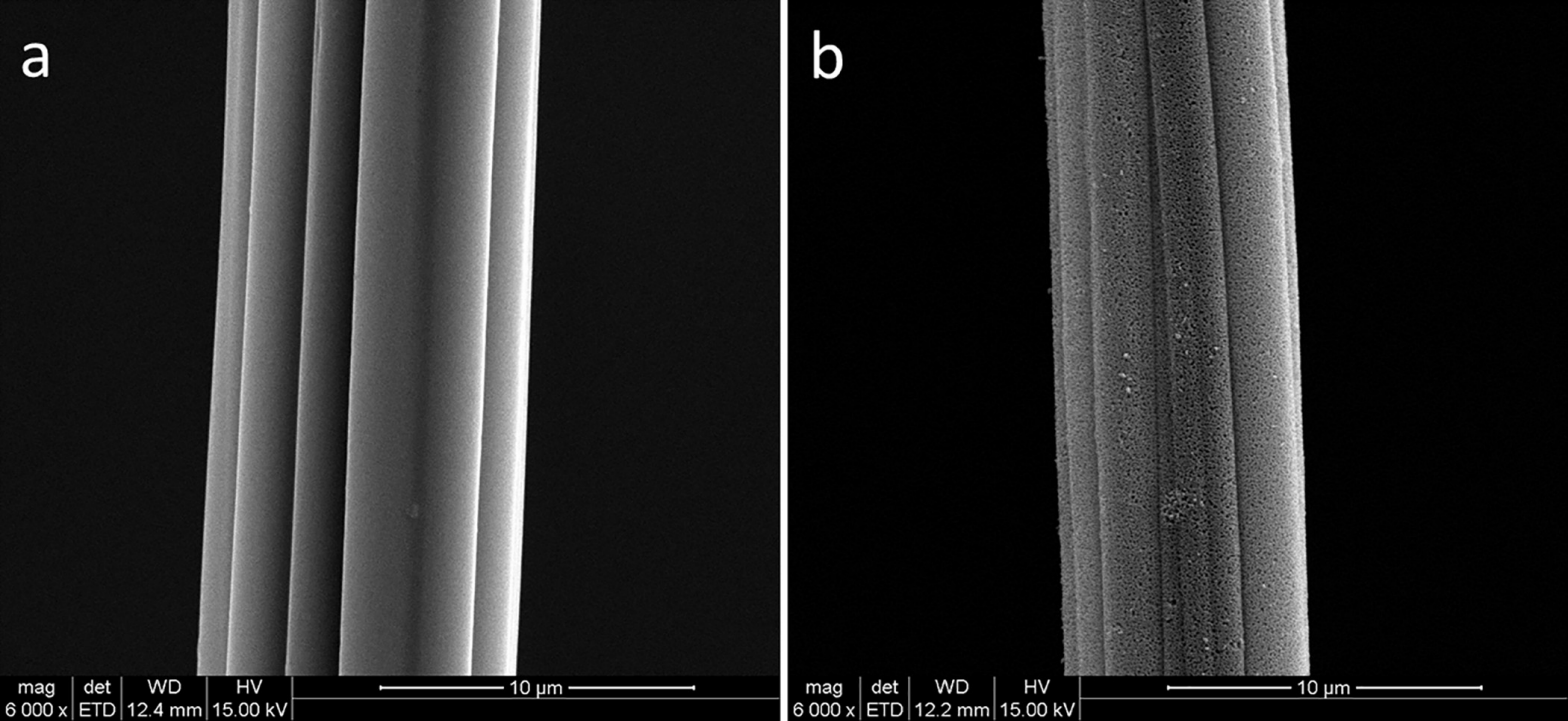
Scanning electron microscopy (SEM) of untreated and carbonized bamboo fiber pretreated with CaCl_2_ at 1000°C (a: Control; b: Carbonized BFCa_5_ fiber at 1000°C).

The SEM images of control and BFCa_5_ carbonized samples at 1000°C are shown in Fig 3: the key difference between the samples is that the control samples have a smooth surface (Fig 3A) whereas many voids are seen on the surface of the carbonized samples (Fig 3B).

The BFCa_5_ retained the original shape of the fibers like the control. The presence of inorganic salts in cellulose influences its flammability by modifying the ability of cellulose to produce the volatile tar (levoglucosan). The presence of salts favors the formation of anhydrocellulose and its subsequent decomposition products, thereby reducing the amount of levoglucosan formed [33]. The FTIR analysis indicated that dehydration of cellulose begins at 210°C, resulting in the formation of anhydrocellulose [34] under the catalytic effect of Ca^2+^; anhydrocellulose is considerably more reactive at elevated temperature and rapidly undergoes numerous further reactions that result in the formation of the main gaseous products (CO, CO_2_, H_2_O, CH_4_, and H_2_) and residual char. Thus, the CaCl_2_ treatment not only improved the carbon yield, but also led to the production of porous fiber. We speculated that the pores on the fibers resulted from the creation of gaseous products during carbonization. In other words, pretreatment with 5% CaCl_2_ at higher temperature is thought to improve the carbonization yield and help maintain the original shape of cellulose fibers, and the formation of main gaseous products helps in obtaining the porous structure of fibers.

**Fig 4:**
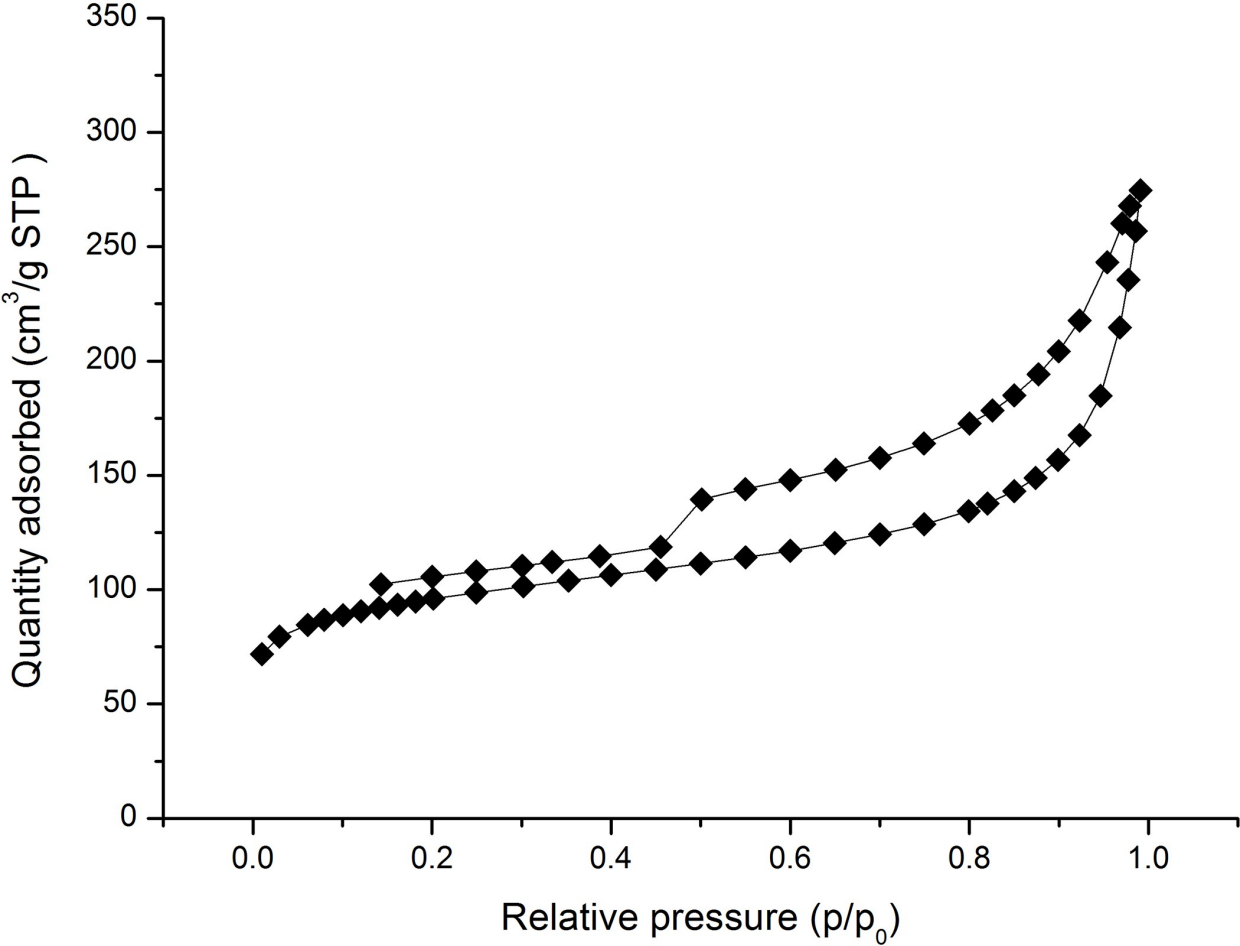
The nitrogen adsorption/desorption isotherms of carbonized bamboo fiber pretreated with CaCl_2_.

The N_2_ adsorption/desorption isotherms are commonly measured to determine the structural properties of solids, especially, porous materials. These measurements provide information about the specific surface area of the material and the size distribution and volume of the pores. The types of pores in the carbonized fiber pretreated with CaCl_2_ were determined by measuring the N_2_ adsorption/desorption isotherms (Fig 4). The CaCl_2_ pretreatment significantly changed the surface structure of carbonized fibers compared to that in the control sample, and the pretreated carbonized fiber showed BET specific surface area of 331.32 m^2^/g. The N_2_ adsorption/desorption isotherms of pretreated carbonized bamboo fiber in the Fig 4 exhibited a typical Type-IV isotherm (isotherm with hysteresis loop) feature according to the IUPAC [35].

For a porous material that present a Type IV isotherm, gases are adsorbed inside the pores of the material and initially form a monolayer. As more molecules are absorbed a multilayer is formed and eventually absorption transitions to capillary condensation [36]. The shape of the hysteresis loop could be used to analyze the morphology of pore shape in carbonized fibers [35, 37]. The Type IV isotherm indicates that our carbonized bamboo fibers contain both meso- and macropores [35]. For the initial part of the isotherm, P/P_0_ range of 0–0.1, the adsorption is restricted to a thin layer of adsorbate on the pore walls and this was interpreted as indicating the formation of a continuous monomolecular layer of gas on the surface. As pressure increases, the isotherm is approximately linear corresponding to the absorption of additional layers of molecules. As the adsorption proceeds, the slop increase at higher elevated pressures indicates an increased uptake of adsorbate as the pores are being filled. When the saturation vapor pressure P/P_0_ becomes 1, wide pores appear and condense at the saturation vapor pressure. As the pressure is lowered, the process reverses but follows a typical hysteresis path, i.e., the slope of the path is similar to that of absorption, but at higher value [38]. This type of hysteresis loops is usually observed in open pores, which contain mainly inkbottle-shaped pores and a small amount of parallel-plate pores or cylindrical pores [35, 39].

**Fig 5:**
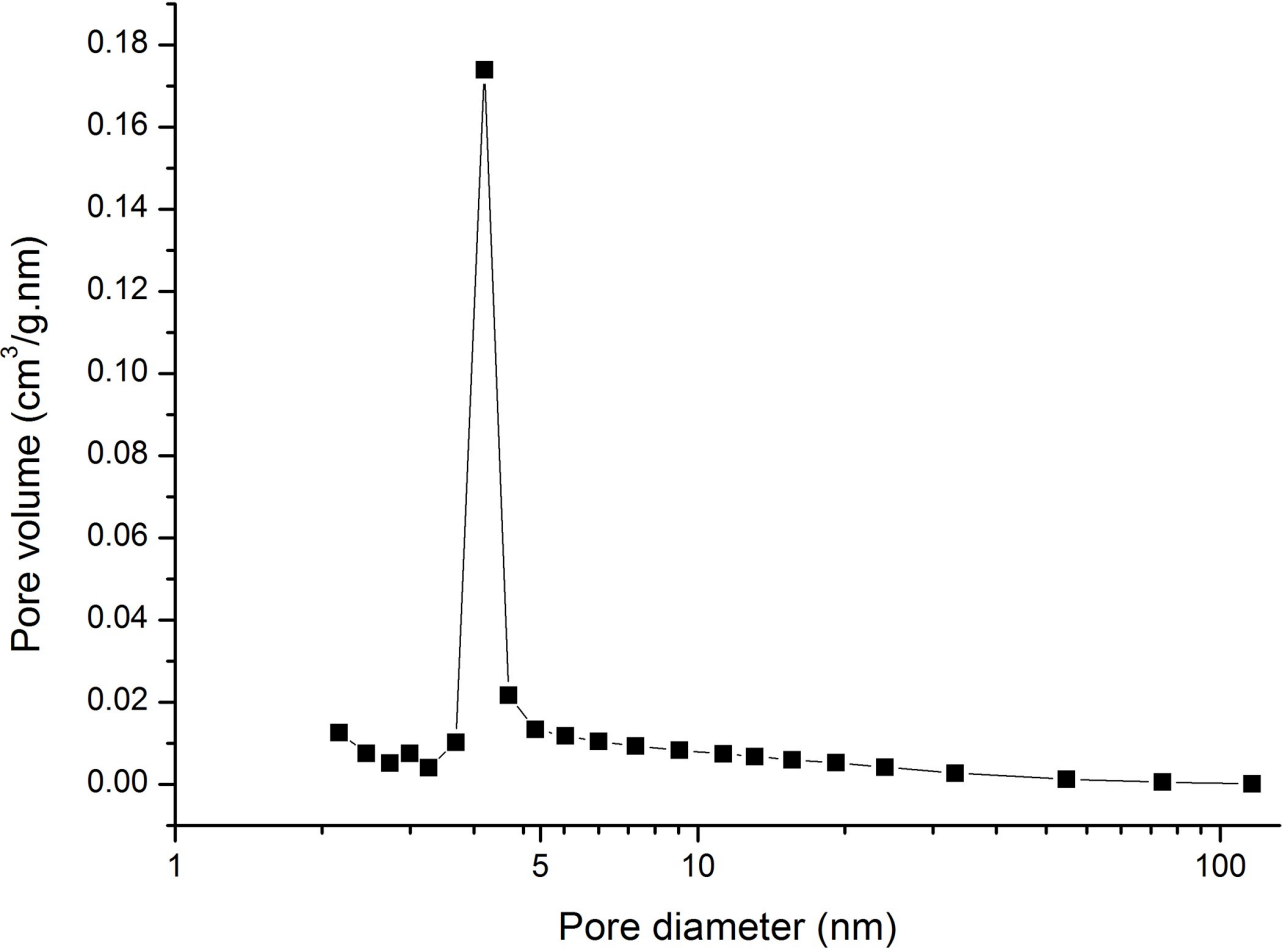
Pore volume distribution with pore sizes obtained from the adsorption branch of isotherms by using the Barrett–Joyner–Halenda (BJH) method.

The distribution of pore diameter of carbonized bamboo fiber calculated according to the BJH method is shown in Fig 5. Considering the characterization of Type IV isotherm with inkbottle-shaped pores, the wide portion of the pores might not be able to evaporate until the narrow neck empties during desorption. Therefore, the pore size distribution should be determined using the adsorption branch of the isotherm [40].

The pore size distribution plot analyzed based on BJH indicated that the pore distribution ranged within 1.7–300 nm, reflecting the distribution of carbonized fibers to have a multipore character with a major peak at 3.91 nm. The pore sizes of the carbonized bamboo fiber showed a wide distribution between 2.06 and 31.07 nm, indicating the presence of numerous mesopores. The BJH adsorption cumulative volume of pores between 1.7 and 300 nm diameter was 0.3307 cm^3^/g, and the BJH adsorption average pore diameter was 13.6440 nm. The pretreatment with CaCl_2_ was highly effective in increasing the BET area and the number of mesopores. Thus, the pretreatment with 5% CaCl_2_ could promote the porous structure formation in carbon fibers.

## Conclusions

(1) Bamboo fiber pretreated with CaCl_2_ shows a decrease of the temperature of the maximum rate of weight lossi to <300°C and a decrease of the overall weight loss, whereas increase in the formation of char residue. The char residue is increased to 13.04%, 31.89%, and 36.92% at 800°C after pretreatment with 1%, 5%, and 10% CaCl_2_.

(2) BFCa_5_ is expected to undergo dehydration since the combined effect of oxygen-rich atmosphere and CaCl_2_ catalysis begins from 210°C and the remarkable decomposition of cellulose would initiate at 250°C considering that the bands at 1024 and 1162 cm^-1^ decreased remarkably and even disappeared, whereas those at 1610 and 1510 cm^-1^ appeared simultaneously, which represented the skeleton stretching vibrations of the benzene ring.

(3) Pretreatment with 5% CaCl_2_ could improve the carbonization yield and help maintain the original shape of the cellulose fibers. Furthermore, production of the main gas product would help in the formation of the porous structure of fibers, which exhibit a typical Type-IV isotherm with the BET specific surface area of 331.32 m^2^/g and BJH adsorption average pore diameter of 13.6440 nm.
